# Willingness of people living with HIV to receive a second COVID-19 booster dose: a multicenter cross-sectional study in China

**DOI:** 10.3389/fpubh.2023.1227277

**Published:** 2023-08-23

**Authors:** Xinquan Lan, Bin Su, Shijie Liang, Maohe Yu, Ying Qiao, Li Wang, Moxin Song, Yuxiao Wang, Junjie Xu

**Affiliations:** ^1^Clinical Research Academy, Peking University Shenzhen Hospital, Peking University, Shenzhen, China; ^2^Department of Epidemiology, School of Public Health, China Medical University, Shenyang, China; ^3^Beijing Key Laboratory for HIV/AIDS Research, Beijing Youan Hospital, Capital Medical University, Beijing, China; ^4^Department of Infection, Zhengzhou Center for Disease Control and Prevention, Zhengzhou, China; ^5^Department of AIDS/STD Control and Prevention, Tianjin Center for Disease Control and Prevention, Tianjin, China; ^6^Department of Infection, The Second Hospital of Hohhot, Hohhot, China; ^7^Department of Infection, Heilongjiang Provincial Hospital, Harbin, China; ^8^Clinical Research Academy, Peking University Shenzhen Hospital, Peking University-The Hong Kong University of Science and Technology Medical Center, Shenzhen, China

**Keywords:** COVID-19 vaccines, PLWH, health belief model, willingness, booster dose

## Abstract

**Background:**

The coronavirus disease 2019 (COVID-19) pandemic has significantly affected the global population, with People Living with HIV (PLWH) being particularly vulnerable due to their compromised immune systems. Although vaccination is a crucial preventative measure against the severe acute respiratory syndrome coronavirus 2 (SARS-CoV-2) virus, little is understood about the willingness of PLWH to receive a second COVID-19 booster dose and the factors that may influence this decision. This study investigates the willingness of PLWH in China to receive a second COVID-19 booster dose and its influencing factors, comparing these with a group of healthy individuals.

**Methods:**

A multicenter cross-sectional study was conducted across five Chinese cities, namely, Beijing, Tianjin, Zhengzhou, Hohhot, and Harbin. Participants were recruited through five community-based organizations. Data were collected *via* participant self-administered questionnaires included demographic information, willingness to receive a second COVID-19 booster dose, and knowledge about HIV and COVID-19 vaccination. Factors influencing vaccination willingness were identified using multivariable logistic regression analyzes.

**Results:**

A total of 156 PLWH and 151 healthy individuals were included in the study. After adjusting for potential confounders, it was found that PLWH demonstrated a lower willingness to receive a second COVID-19 booster dose compared to healthy individuals (77.6% vs. 88.7%, *p* = 0.009). Lower willingness was associated with HIV positive status (Adjusted Odds Ratio [AOR]: 0.39, 95%CI: 0.20, 0.75), perceived barriers (AOR: 0.05, 95%CI: 0.01, 0.26), and perceived severity (AOR: 0.32, 95%CI: 0.12, 0.90).

**Conclusion:**

PLWH in China demonstrated a lower willingness to receive a second COVID-19 booster dose compared to healthy individuals. The findings suggest that perceptions and understanding of the COVID-19 vaccination and its necessity for protection against SARS-CoV-2 could influence this willingness. Efforts should be made to strengthen and disseminate knowledge about HIV and COVID-19 vaccinations among this population. In addition, developing interventions and policies that target specific subgroups and address misconceptions about vaccination could be instrumental in improving vaccination rates among PLWH.

## 1. Introduction

The coronavirus disease 2019 (COVID-19), caused by the severe acute respiratory syndrome coronavirus 2 (SARS-CoV-2), has escalated into a global pandemic, with over 750 million infections and 6.8 million deaths recorded as of February 1, 2023 (1). The impacts are particularly severe for specific populations, including people living with HIV (PLWH), who have been shown to have a higher risk of SARS-CoV-2 infection, serious illness, hospitalization, and death than the general population (2–6).

COVID-19 vaccines have been recognized as one of the most effective methods for preventing infection with SARS-CoV-2 and its variants (7, 8). Vaccination has significantly reduced the risk of SARS-CoV-2 infection and severe COVID-19 disease outcomes in PLWH (9, 10). However, the rise of new SARS-CoV-2 variants has necessitated booster doses of COVID-19 vaccines in some countries. Initial studies indicated the effectiveness of the first COVID-19 booster dose among PLWH against new variants such as Delta and Omicron (11–13). However, other research suggests that immunogenicity and the effectiveness of preventing severe outcomes with the first COVID-19 booster dose among PLWH may diminish over time, especially concerning the Omicron variant (14).

Given the potential decrease in immunogenicity and effectiveness over time, PLWH should receive a second COVID-19 booster dose at an appropriate time. Further research indicates enhanced immunogenicity and safety with the second COVID-19 booster dose in PLWH (15). In response to these findings, several countries, including China, now recommend a second booster dose for PLWH, along with other key populations such as individuals over the age of 60, high-risk groups, those with underlying health conditions, and particularly immunocompromised individuals (16–18).

In the past, significant hesitancy was observed among PLWH in China regarding full-dose COVID-19 vaccination. The vaccination coverage among this group was significantly lower than the international average for the PLWH population (6.2% vs. 63.5%) (19, 20). With the current promotion of the second COVID-19 booster dose both in China and globally, understanding the vaccination willingness of PLWH and exploring the relevant influencing factors have profound theoretical and practical implications for developing and promoting vaccination strategies.

According to the World Health Organization (WHO), it is still necessary for individuals who have previously been infected with SARS-CoV-2 to receive a second COVID-19 booster dose (21). However, only Uganda has reported vaccination rates and willingness to receive the first COVID-19 booster doses among PLWH (22). Given the significant differences in COVID-19 vaccination types and perceptions across countries, the results from other contexts cannot directly guide COVID-19 vaccination strategies for PLWH in China.

Previous studies have highlighted the concern about vaccine side effects as a significant factor influencing the hesitation of PLWH to receive the COVID-19 vaccine. Whether side effects after the first COVID-19 booster dose influence the willingness to receive the second booster dose remains unclear. Addressing these knowledge gaps would offer valuable insights to guide the administration of the second COVID-19 booster dose among PLWH.

The HBM is one of the most extensively utilized theories for understanding health and illness behaviors. The model is predicated on the understanding that a person’s belief in a personal threat of an illness or disease and belief in the effectiveness of the recommended health behavior or action will predict the likelihood that the person will adopt the behavior. The HBM has been previously employed to analyze COVID-19 complete vaccination willingness and behavior among cancer patients and PLWH, as well as in health education activities related to vaccine promotion (23, 24). Although applying HBM to COVID-19 vaccination could enhance our understanding of this health behavior, there is still a gap in the literature, particularly about COVID-19 booster vaccination among PLWH.

In this study, we developed a questionnaire based on the HBM to conduct an anonymous survey among the PLWH population in mainland China. This study aims to provide a theoretical basis for guiding the effective adjustment and implementation of vaccination strategies in our country and other nations in response to the continuously evolving disease situation.

## 2. Materials and methods

### 2.1. Study design and objective

This cross-sectional survey is derived from a registered prospective cohort study (the Chinese Clinical Trial Registry.ChiCTR2200058989). The prospective cohort study aimed to assess changes in immunogenicity and adverse reactions within 6 months following the first COVID-19 booster dose in China among PLWH. The prospective cohort study initially recruited both PLWH and healthy individuals in five Chinese cities (Beijing, Tianjin, Zhengzhou, Hohhot, and Harbin), with participant recruitment and selection criteria described in our previous work (25). Based on the cohort study, we further conducted a cross-sectional survey from December 2021 to March 2022. The present study has been approved by the Ethics Committee of Peking University Shenzhen Hospital (No. 2021-094).

### 2.2. Participants

In this study, the inclusion criteria for participants included: (1) aged between 18 and 65 years, (2) no history of SARS-CoV-2 infection, (3) having received full immunization (two doses of COVID-19 inactivated vaccine) and the first COVID-19 inactivated booster dose, (4) the second COVID-19 booster dose has not been vaccinated yet, and (5) willingness to participate in the study activities and having signed written informed consent. The HIV infection status was preliminarily self-reported by participants before attending this site study. We re-identified the HIV serostatus for PLWH using the Abbott ARCHITECT HIV Ag/Ab Combo assay, which has high sensitivity and specificity (S/CO ≥ 1.0, Reactive) at the study site. The exclusion criteria were: (1) interviewees with severe hearing loss, visual impairment, or intellectual disability and (2) major mental illness (schizophrenia or bipolar disorder) or neurocognitive impairment as assessed by the clinician.

### 2.3. Study procedures

PLWH was recruited from five community-based organizations that collaborated with HIV clinical service providers and offered services to PLWH, one in each city. Recruitment advertisements were disseminated through WeChat public accounts, a widely used social media platform in China. Then, interested PLWH contacted project staff via social media and were briefly informed of the study’s purpose and procedure. Potential PLWH participants and the healthy control population received a detailed informed consent form. Upon signing, they were screened using inclusion criteria and a free HIV test through the HIV rapid test kit. Eligible HIV-negative individuals were also invited to participate in the study. Investigators issued an anonymous questionnaire through the online survey platform (Golden Data) at the prevaccination (before 2–4 weeks of receiving the first COVID-19 booster dose) and the fourth-week follow-up of the prospective cohort study to understand their feelings and willingness after the first COVID-19 booster dose. Questionnaires that did not meet the length (less than 100 s) to fill in the questionnaire and had logical errors (For instance: the time of COVID-19 vaccination was before the occurrence of COVID-19) were excluded.

### 2.4. Questionnaire

The questionnaire used in this survey consisted of five sections: Socio-demographic characteristics and health status; Adverse reactions after vaccination; Willingness to receive the second COVID-19 booster dose; HBM project; HIV-related information and immunization status.

To ensure effectiveness, all questions were constructed and evaluated by an expert team (including two public health experts and an epidemiologist specializing in infectious diseases).

In the HBM section, we set up 16 items across six dimensions, including perceived susceptibility (3 items), perceived severity (3 items), perceived harm (1 item), perceived benefits (2 items), behavioral cues (1 item), and self-efficacy (1 item). The score for each item ranged from 1 to 5, allocated to “strongly disagree, ““disagree, ““neutral, ““agree, “and “strongly agree.” The scale’s reliability was verified by Cronbach’s α coefficient (*α* = 0.835).

HIV-related information and immunization status included current HIV infection status, HIV infection time, ART conditions, the latest testing results of HIV viral load, and CD4 + T cell absolute count.

The questionnaire was anonymous, with a unique 6-digit number for each participant to protect privacy. A master list with identifiable information was saved on the principal investigator’s computer with password protection, accessible only to the principal investigator, and the data were encrypted and regularly backed up to prevent data loss or unauthorized access.

### 2.5. Sample size

This study aimed to evaluate Chinese PLWH’s willingness to vaccinate with the second COVID-19 booster dose relative to healthy individuals. Based on the results of published peer-reviewed studies in Greece, Italy, and China, it was estimated that the acceptance rate of the second COVID-19 booster dose among PLWH is 70%. The acceptance rate among the healthy control group is 85% (26–28). The confidence level of 1−*α* = 95% and the test efficacy 1−*β* = 0.8 were specified. After considering a 10% dropout rate, 270 participants were required, with the PLWH group and healthy individuals group allocated in a 1:1 ratio. The sample size was calculated using Power Analysis and Sample Size software (version 15.0.5).

### 2.6. Statistical analysis

For continuous variables, normality was assessed by the Shapiro–Wilk test. Variables conforming to normal distribution were analyzed using the t-test method, and for non-conforming variables, the Mann–Whitney test method was used. For categorical variables, the chi-square/Fisher method was used. A logistic regression analysis was performed to investigate the factors influencing vaccination willingness. First, a binary logistic regression analysis was performed for demographic characteristics information to obtain variables with *p* < 0.05. After that, the variables with *p* < 0.05 were added to the multivariable logistic regression analysis to correct for the bias introduced by background information. Associations between the independent variables of interest (i.e., variables at the individual, HBM project, and HIV-related information and immunization status) and the dependent variables were assessed by adjusted odds ratios (AORs) and 95% confidence intervals. Each AOR was obtained by fitting a logistic regression model involving an independent variable of interest and all significant background characteristics. All statistical analyzes were performed using SPSS software (version 25.0 IBM Corp., Armonk, NY). A two-tailed *p* value of less than 0.05 was considered statistically significant.

## 3. Results

### 3.1. Background characteristics

**Figure 1:**
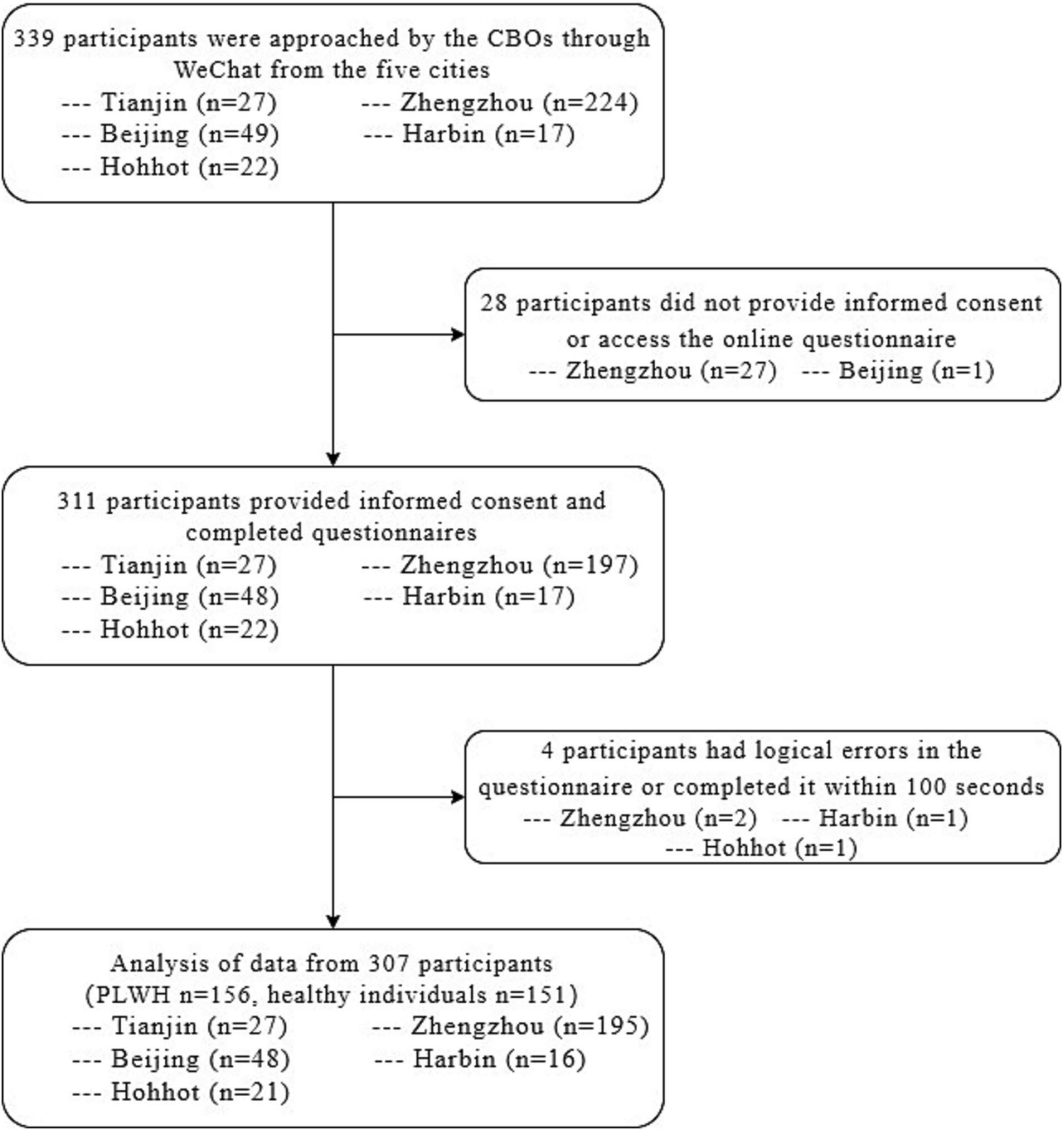
Data collection procedures in the study.

A total of 339 participants aged between 18 and 65 years old were approached for participation. Thirty-two participants were excluded from the study for four reasons: non-provision of informed consent, failure to complete the online questionnaire, presence of logical errors in the questionnaire responses, and inappropriate completion time. Of the remaining 307 participants, 50.81% were PLWH (156/307), and 49.19% were healthy individuals (151/307) (Figure 1).

All participants had been vaccinated with the preliminary schedule of two doses of inactivated COVID-19 before recruitment for this study. On average, they received the first COVID-19 booster dose 203 days after receiving the initial two doses. The PLWH group had a significantly higher proportion of males (96.2% vs. 86.8%, *p* = 0.003) and individuals aged 30–45 (56.4% vs. 46.4%, *p* = 0.045) than the healthy individual group. Conversely, the healthy individual group had a significantly lower proportion of single/divorced/widowed (56.3% vs. 82.7%, *p* < 0.001) and engaged in full-time work (43.7% vs. 67.3%, *p* = 0.047) than the PLWH group. Regarding HIV-related information and immunization status, 84.6% of PLWH had been infected with HIV for over 2 years, with the majority (92.9%) receiving ART. 51.9% of PLWHs reported that their last HIV viral load test was undetectable, and 78.8% of PLWHs reported that their last CD4+ T cell count was over 200 cells/mm^3^. There were no significant differences (*p* > 0.05) between the PLWH and healthy individual group regarding education level, monthly income, or prevalence of chronic underlying diseases. More details of the background characteristics can be found in Table 1.

**Table 1:** Sociodemographic characteristics of 307 PLWH and healthy individuals.

	Total *n* (%) (*N* = 307)	PLWH *n* (%) (*N* = 156)	Non-PLWH *n* (%) (*N* = 151)	*p* value
*Sociodemographic characteristics*				
Age group (years)				
18–29	71 (23.1)	38 (24.4)	33 (21.9)	0.045
30–45	158 (51.5)	88 (56.4)	70 (46.4)	
46–59	68 (22.1)	28 (17.9)	40 (26.5)	
≥60	10 (3.3)	2 (1.3)	8 (5.3)	
Gender				
Male	281 (91.5)	150 (96.2)	131 (86.8)	0.003
Female	26 (8.5)	6 (3.8)	20 (13.2)	
Education level				
Junior high or below	44 (14.3)	18 (11.5)	26 (17.2)	0.249
Senior high or equivalent	98 (31.9)	48 (30.8)	50 (33.1)	
College and above	165 (53.7)	90 (57.7)	75 (49.7)	
Relationship status				
Single/divorced/widowed	214 (69.7)	129 (82.7)	85 (56.3)	<0.001
Married	93 (30.3)	27 (17.3)	66 (43.7)	
Employment status				
Full-time	190 (61.9)	105 (67.3)	66 (43.7)	0.047
Part-time/self-employed/unemployed/retired/students	117 (38.1)	51 (32.7)	85 (56.3)	
Monthly income (CNY)				
<3,000	71 (23.1)	33 (21.2)	38 (25.2)	0.692
3,000–6,999	181 (59.0)	95 (60.9)	86 (57.0)	
≥7,000	55 (17.9)	28 (17.9)	27 (17.9)	
*Presence of chronic disease conditions (not including HIV)*				
Yes	24 (7.8)	14 (9.0)	10 (6.6)	0.443
No	283 (92.2)	142 (91.0)	141 (93.4)	
Type of chronic diseases				
Diabetes mellitus	3 (1.0)	2 (1.3)	1 (0.7)	0.581
Hypertension and/or hyperlipidaemia	10 (3.3)	6 (3.8)	4 (2.6)	0.555
Chronic cardiovascular diseases^a^	3 (1.0)	2 (1.3)	1 (0.7)	0.581
Chronic respiratory diseases^b^	4 (1.3)	3 (1.9)	1 (0.7)	0.330
Other chronic diseases^c^	8 (2.6)	5 (3.2)	3 (2.0)	0.503
*HIV related characteristics*				
Time since HIV diagnosis (years)			N/A	
≤1		13 (8.3)		
2–5		76 (48.7)		
>5		56 (35.9)		
Not sure		11 (7.1)		
On antiretroviral therapy			N/A	
Yes		145 (92.9)		
No		11 (7.1)		
HIV viral load in the most recent episode of testing (copies/mL)			N/A	
Undetectable (<50)		81 (51.9)		
Detectable (≥50)		45 (28.8)		
Not sure		30 (19.2)		
CD4+ T cell count in the most recent episode of testing (cells/mm^3^)			N/A	
>500		54 (34.6)		
200–500		69 (44.2)		
<200		6 (3.8)		
Not sure		27 (17.3)		

N/A, not applicable; CNY, Chinese yuan.

a Chronic cardiovascular disease include chronic heart failure, coronary heart disease, congenital heart disease and valvar heart disease.

b Chronic respiratory diseases include chronic obstructive emphysema disease, asthma, chronic cor pulmonale and chronic respiratory failure.

c Other chronic diseases include malignant tumors, Immune thrombocytopenia, chronic hepatitis B, gout, etc.

### 3.2. Vaccination intention and adverse reactions

A significant difference was observed in the willingness to receive the second COVID-19 booster dose between the PLWH group and the healthy individual group (77.6% vs. 88.7%, *p* = 0.009).

Regarding adverse reactions within 1 month of receiving the first COVID-19 booster dose, 5.1% of the PLWH group and 2.0% of the healthy individual group reported adverse reactions. The primary adverse reactions were local, with 4.5% of the PLWH group and 2.0% of the healthy individual group experiencing them. In the PLWH group, the main complaint was pain at the inoculation site (4.6%), while in the healthy individual group, the main complaint was redness at the inoculation site (2.0%). No significant difference was found between the two groups in the incidence of adverse reactions, local adverse reactions, and systemic adverse reactions (*p* > 0.05). Table 2 presents the specific details of the adverse reactions.

**Table 2:** Adverse reaction after the first COVID-19 booster dose and the willingness regarding the second COVID-19 booster dose (*N* = 307).

	Total *n* (%) (*N* = 307)	PLWH *n* (%) (*N* = 156)	Non-PLWH *n* (%) (*N* = 151)	*p* value
*Adverse reaction*				
Adverse reactions within one month of the first COVID-19 vaccine booster dose	11 (3.6)	8 (5.1)	3 (2.0)	0.139
Local adverse reactions	10 (3.3)	7 (4.5)	3 (2.0)	0.362
Pain	6 (2.0)	4 (2.6)	2 (1.3)	0.685
Redness	4 (1.3)	1 (0.6)	3 (2.0)	0.365
Pruritus	4 (1.3)	3 (1.9)	1 (0.7)	0.623
Rash	2 (0.7)	2 (1.3)	0 (0.0)	0.498
Induration	1 (0.3)	1 (0.6)	0 (0.0)	>0.999
Systematic adverse reactions	1 (0.3)	1 (0.6)	0 (0.0)	>0.999
Headache	1 (0.3)	1 (0.6)	0 (0.0)	>0.999
*Willingness to get the fourth dose of COVID-19 vaccine*				
Whether you will receive the second COVID-19 vaccine booster dose				
Very unlikely/unlikely/neutral	52 (16.9)	35 (22.4)	17 (11.3)	0.009
Likely/very likely	255 (83.1)	121 (77.6)	134 (88.7)	

### 3.3. Health belief model measures

Table 3 presents the attitudes of all participants regarding the six primary dimensions of the HBM and the specific items in each dimension. In five dimensions—perceived benefit, perceived susceptibility, perceived severity, action clues, and self-efficacy—the PLWH group scored significantly lower than the healthy individual group (*p* < 0.001). Conversely, in the dimension of perceived barriers, the PLWH group scored significantly higher than the healthy individual group (7.1% vs. 1.3%, *p* = 0.004). These results suggest that the PLWH group may face more obstacles and be less motivated to receive the COVID-19 booster than the healthy individuals group.

**Table 3:** HBM items: perceived susceptibility, perceived severity, perceived benefits, perceived barriers, cues of action, and self-efficacy (*N* = 307).

	Total *n* (%) (*N* = 307)	PLWH *n* (%) (*N* = 156)	Non-PLWH *n* (%) (*N* = 151)	*p* value
**Perceived benefits**				
*Feelings after the COVID-19 vaccination booster (the third dose)*				
Benefits of COVID-19 vaccination booster compared to expectations				
More (some more/a lot more)	194 (63.2)	88 (56.4)	106 (70.2)	0.033
No change	101 (32.9)	62 (39.7)	39 (25.8)	
Less (less/much less)	12 (3.9)	6 (3.8)	6 (4.0)	
Physical status after COVID-19 vaccination booster compared to expectations				
Good (better/much better)	125 (40.7)	44 (28.2)	81 (53.6)	<0.001
No change	173 (56.4)	105 (67.3)	68 (45.0)	
Poor (worse/much worse)	9 (2.9)	7 (4.5)	2 (1.3)	
**Perceived barriers**				
Adverse effects (adverse events or side effects) of COVID-19 vaccine booster compared to expected				
More (some more/a lot more)	13 (4.2)	11 (7.1)	2 (1.3)	0.004
No change	127 (41.4)	72 (46.2)	55 (36.4)	
Less (less/much less)	167 (54.4)	73 (46.8)	94 (62.3)	
**Perceived susceptibility**				
*Positive attitudes toward COVID-19 vaccine booster dose (agree/strongly agree)*				
Receiving a booster dose can maintain your antibody level and strengthen the protection against COVID-19	226 (73.6)	96 (61.5)	130 (86.1)	<0.001
A booster dose is highly effective in protecting you from COVID-19 variants of concern (e.g., Omicron)	237 (77.2)	110 (70.5)	127 (84.1)	0.006
There is a sufficient supply of COVID-19 vaccine in China to strengthen the vaccination work for many times	251 (81.8)	118 (75.6)	133 (88.1)	0.007
**Perceived severity**				
*Negative attitudes toward COVID-19 vaccine booster dose (agree/strongly agree)*				
The side effects of COVID-19 vaccine booster dose are more severe	22 (7.2)	8 (5.1)	14 (9.3)	<0.001
Multiple vaccinations to strengthen the needle will bring unknown long-term health risks	20 (6.5)	6 (3.8)	14 (9.3)	<0.001
The duration of protection of COVID-19 vaccine booster dose is shorter	31 (10.1)	12 (7.7)	19 (12.6)	<0.001
**Cues of action**				
People who are important to you (e.g., family member, doctors) would support you to receive a booster dose	234 (76.2)	100 (64.1)	134 (88.7)	<0.001
**Self-efficacy**				
Receiving a COVID-19 vaccine booster dose is easy for you if you want to	240 (78.2)	107 (68.6)	133 (88.1)	<0.001

### 3.4. Factors associated with willingness to receive the second COVID-19 booster dose

Table 4 shows the results from the univariate analysis. Notably, willingness to receive a second COVID-19 booster dose was higher among those aged 18 to 29 years (90.1%) compared to those aged 30 years and older (79.1, 88.2, 60.0%). Similarly, those with a monthly income of 3,000 to 6,999 Yuan were more willing to receive the booster dose (87.8%) compared to those earning less than 3,000 Yuan (77.5%) and more than 7,000 Yuan (74.5%).

**Table 4:** Univariate logistic regression of participants’ sociodemographic characteristics (*N* = 307).

Variable	Whether you will receive the second COVID-19 booster dose (the fourth dose)		
	Vaccine acceptance *n*/*N* (%)	Odds Ratio (95% CI)	*p* value
*Sociodemographic characteristics*			
Age group (years)			
18–29	64/71 (90.1)	Reference	
30–45	125/158 (79.1)	0.41 (0.17–0.99)	0.047
46–59	60/68 (88.2)	0.82 (0.28–2.40)	0.718
≥60	6/10 (60.0)	0.16 (0.04–0.73)	0.017
Gender			
Female	21/26 (80.8)	Reference	
Male	234/281 (83.3)	1.19 (0.43–3.30)	0.745
Education level			
Junior high or below	35/44 (79.5)	Reference	
Senior high or equivalent	88/98 (89.8)	2.26 (0.85–6.04)	0.103
College and above	132/165 (80.0)	1.03 (0.45–2.35)	0.947
Relationship status			
Single/divorced/widowed	174/214 (81.3)	Reference	
Married	81/93 (87.1)	1.55 (0.77–3.12)	0.217
Employment status			
Part-time/self-employed/unemployed/retired/students	94/117 (80.3)	Reference	
Full-time	161/190 (84.7)	1.36 (0.74–2.48)	0.320
Monthly income (Yuan)			
<3,000	55/71 (77.5)	Reference	
3,000–6,999	159/181 (87.8)	2.10 (1.03–4.29)	0.041
≥7,000	41/55 (74.5)	0.85 (0.37–1.94)	0.703
Presence of chronic disease conditions (not including HIV)			
No	236/283 (83.4)	Reference	
Yes	19/24 (79.2)	0.76 (0.27–2.13)	0.597

Statistically significant values are identified in boldface (*α* < 0.05). CI: confidence interval.

After adjusting for statistically significant sociodemographic characteristics, the outcome of lower willingness to receive the second booster dose was independently associated with HIV positivity (AOR: 0.39, 95%CI: 0.20, 0.75), perceived barriers (indicating the expectation of more adverse effects from the COVID-19 vaccine booster) (AOR: 0.05, 95%CI: 0.01, 0.26), and perceived severity (referring to negative attitudes toward the COVID-19 vaccine booster dose) (AOR: 0.32, 95%CI: 0.12, 0.89).

Conversely, a higher inclination towards receiving the second booster dose was associated with perceived benefits (indicating the expectation of more benefits from the COVID-19 vaccine booster) (AOR: 18.57, 95%CI: 4.02, 85.83) and (referring to better physical status after the vaccination) (AOR: 33.37, 95%CI: 4.22, 263.91). Furthermore, consistent with perceived benefits, a stronger inclination towards receiving the second booster dose showed positive correlations (AOR > 1 for all aforementioned variables) with perceived susceptibility, cues to action, self-efficacy, and detectable HIV viral load. Detailed information (e.g., AOR and 95% CI) can be referenced in Table 5.

**Table 5:** Univariate and multivariable analysis of factors associated with willing to receive the second COVID-19 booster dose (*N* = 307).

Variable	Willing to receive the second COVID-19 booster dose			
	OR (95% CI)	*p* value	AOR (95% CI)	*p* value
HIV infection status				
Negative	Reference		Reference	
Positive	0.44 (0.23–0.82)	0.010	0.39 (0.20–0.75)	0.005
HIV viral load in the most recent episode of testing (copies/mL)				
Undetectable (<50)	Reference		Reference	
Detectable (≥50)	5.55 (1.56–19.71)	0.008	4.98 (1.35–18.37)	0.016
Not sure	0.93 (0.37–2.32)	0.868	0.77 (0.28–2.14)	0.622
CD4+ T cell count in the most recent episode of testing (cells/mm^3^)				
>500	Reference		Reference	
200–500	1.06 (0.11–9.92)	0.963	1.22 (0.13–11.91)	0.865
<200	0.44 (0.05–4.02)	0.463	0.59 (0.06–5.83)	0.651
Not sure	0.70 (0.07–7.20)	0.764	0.82 (0.08–8.81)	0.867
**Adverse reaction**				
Adverse reactions within one month of the first COVID-19 booster dose				
No	Reference		Reference	
Yes	0.92 (0.19–4.36)	0.911	0.65 (0.13–3.29)	0.603
**Perceived benefits**				
*Feelings about the first COVID-19 booster dose*				
Benefits of COVID-19 vaccination booster compared to expectations				
Less (less/much less)	Reference		Reference	
No change	0.70 (0.20–2.49)	0.584	0.81 (0.22–2.98)	0.754
More (some more/a lot more)	15.67 (3.68–66.76)	<0.001	18.57 (4.02–85.83)	<0.001
Physical status after COVID-19 vaccination booster compared to expectations				
Poor (worse/much worse)	Reference		Reference	
No change	1.34 (0.32–5.58)	0.687	1.30 (0.30–5.60)	0.729
Good (better/much better)	30.75 (4.30–220.04)	0.001	33.37 (4.22–263.91)	0.001
**Perceived barriers**				
Adverse effects (adverse events or side effects) of COVID-19 vaccine booster compared to expectation				
Less (less/much less)	Reference		Reference	
No change	0.05 (0.02–0.13)	<0.001	0.05 (0.02–0.15)	<0.001
More (some more/a lot more)	0.06 (0.01–0.26)	<0.001	0.05 (0.01–0.26)	<0.001
**Perceived susceptibility**				
*Positive attitudes toward COVID-19 vaccine booster dose*				
Receiving a booster dose can maintain your antibody level and strengthen the protection against COVID-19				
Disagree/strongly disagree/neutrality	Reference		Reference	
Agree/strongly agree	23.26 (10.78–50.21)	<0.001	28.65 (12.27–66.92)	<0.001
A booster dose is highly effective in protecting you from COVID-19 variants of concern (e.g., Omicron)				
Disagree/strongly disagree/neutrality	Reference		Reference	
Agree/strongly agree	18.92 (9.24–38.71)	<0.001	18.77 (8.81–39.99)	<0.001
There is a sufficient supply of COVID-19 vaccine in China to strengthen the vaccination work for many times				
Disagree/strongly disagree/ neutrality	Reference		Reference	
Agree/strongly agree	26.44 (12.55–55.70)	<0.001	33.14 (13.94–78.83)	<0.001
**Perceived severity**				
*Negative attitudes toward COVID-19 vaccine booster dose*				
The side effects of COVID-19 booster dose are more severe				
Disagree/strongly disagree/neutrality	Reference		Reference	
Agree/strongly agree	0.40 (0.16–1.04)	0.060	0.32 (0.12–0.89)	0.030
Multiple vaccinations to strengthen the needle will bring unknown long-term health risks				
Disagree/strongly disagree/neutrality	Reference		Reference	
Agree/strongly agree	0.35 (0.13–0.91)	0.032	0.32 (0.12–0.90)	0.031
The duration of protection of COVID-19 vaccine booster dose is shorter				
Disagree/strongly disagree/neutrality	Reference		Reference	
Agree/strongly agree	1.42 (0.48–4.25)	0.529	1.47 (0.47–4.54)	0.508
**Cues of action**				
People who are important to you (e.g., family member, doctors) would support you to receive a booster dose				
Disagree/strongly disagree/neutrality	Reference		Reference	
Agree/strongly agree	30.35 (13.84–66.56)	<0.001	28.89 (12.93–64.57)	<0.001
**Self-efficacy**				
Receiving a COVID-19 vaccine booster dose is easy for you if you want to				
Disagree/strongly disagree/neutrality	Reference		Reference	
Agree/strongly agree	21.15 (10.25–43.65)	<0.001	19.87 (9.39–42.04)	<0.001

OR, crude odds ratios; AOR, adjusted odds ratios, odds ratios adjusted for significant Sociodemographic characteristics listed in Table 3; CI, confidence interval.

## 4. Discussion

To our knowledge, this is the first multicenter cross-sectional study to explore the willingness of PLWH to receive a second COVID-19 booster dose and its influencing factors in China. Our findings suggest that PLWH were more hesitant to receive a second COVID-19 booster dose than the healthy population. The reasons for this hesitation appear to be multifactorial, with HIV infection status, more significant than expected adverse effects after the first COVID-19 booster dose, and negative attitudes toward the COVID-19 vaccine booster dose being the main factors contributing to vaccine hesitancy. Our findings provide important insights into the willingness of PLWH in China to receive a second COVID-19 booster dose and the associated factors influencing this decision. Moreover, our results could inform both the theoretical framework and practical measures for institutions aiming to understand and address the vaccination intentions of PLWH and the factors influencing them. This, in turn, may assist in designing more effective public health interventions and educational campaigns for PLWH, aiming to boost vaccination coverage and minimize the risk of co-infection and severe clinical outcomes.

Our study observed a lower willingness among PLWH to receive a second COVID-19 booster dose compared to full immunization and the first COVID-19 booster dose reported in the United States, Italy, and Latin America and the Caribbean (78–86.2%) (29–31). One possible explanation for this disparity might be that over time, China’s measures to control COVID-19 have not diminished, yet the prolonged duration of such controls has engendered a sense of fatigue among the population. Consequently, this has led to PLWH beginning to underestimate the pathogenic potential of SARS-CoV-2 and its variants. At the same time, our study corroborates previous findings in healthy individuals in China indicating a high willingness to receive a second COVID-19 booster dose (81.1% vs. 88.7%) (32). However, our study, after adjusting for potential confounders, revealed a significant association between HIV status and vaccine hesitancy for the second COVID-19 booster dose. This persistent vaccine hesitancy among PLWH in China warrants further investigation, despite demonstrated safety and preventative efficacy of the fourth COVID-19 dose and ongoing promotion by relevant health departments (15). It suggests the need for targeted interventions and education to address the factors contributing to this hesitancy.

To date, abundant studies investigating COVID-19 vaccine acceptance among PLWH has generated valuable insights into the underlying influencing factors. For instance, a cross-sectional study demonstrated that negative attitudes towards prime vaccines was associated with the diminished likelihood of vaccine acceptance in China (33). Conversely, individuals with positive perceptions of the prime COVID-19 vaccine exhibited higher rates of acceptance. Furthermore, a positive association between the booster vaccine acceptance and beliefs in the safety, benefits, and accessibility of the booster vaccine was also proved in Uganda (22). Our study further demonstrated that negative attitudes towards vaccines and perceived barriers were both associated with reduced acceptance of the second COVID-19 booster Dose. Similar results were found in immunocompromised cancer patients, which strengthened the necessity of vaccination among specific populations (34, 35).

Our study is the first to investigate the relationship between the willingness of PLWH in China to receive the second COVID-19 booster dose and the six main dimensions of the HBM. These dimensions include perceived susceptibility, perceived severity, perceived benefits, perceived barriers, cues to action, and self-efficacy. Our findings indicate that perceived barriers negatively correlate with vaccine willingness, suggesting fears and misconceptions may dissuade PLWH from receiving the booster dose (36). On the contrary, perceived benefits were positively associated with vaccine willingness, highlighting the potential impact of understanding the benefits of vaccination in promoting vaccine acceptance. Interestingly, we found a positive correlation between perceived susceptibility and vaccine willingness, suggesting that individuals at risk of contracting COVID-19 may be more willing to get vaccinated. However, perceived severity was negatively associated with vaccine willingness, which could indicate that those who perceive COVID-19 as a severe disease may have heightened fears about the safety of vaccines (37). We also noted a positive correlation between self-efficacy and preventive behavior, reinforcing that individual belief in their ability to take preventive measures successfully can influence their willingness to vaccinate (38, 39). Finally, our findings showed a positive correlation between cues to action and vaccine willingness. This implies that support and encouragement from family, friends, and doctors could be critical in promoting vaccination among PLWH (33, 40).

In light of these findings, health departments in China should amplify their efforts to communicate the benefits of the second COVID-19 booster dose. This includes providing clear and reassuring information about the vaccine’s safety, encouraging social support networks to promote vaccination, and fostering a sense of self-efficacy among PLWH. Addressing these factors can reduce vaccine hesitancy and increase the second COVID-19 booster dose uptake among PLWH.

Our multivariable logistic regression analysis revealed that PLWH with a detectable HIV viral load (≥50 copies/mL) demonstrated a higher willingness to receive the second COVID-19 booster dose than those with an undetectable viral load (<50 copies/mL). This result diverges from a US study, which reported a higher willingness to vaccinate among PLWH with an undetectable HIV viral load (29). The discrepancy could be attributed to differences in study design, participant demographics, cultural attitudes towards vaccination, or the methodology of obtaining HIV viral load data. However, the impact of these factors should be further investigated in future studies.

This study has important practical implications, as it found that the willingness of PLWH to receive the second COVID-19 booster dose in China is notably lower than that of the general adult population. It identifies inhibiting factors such as perception barriers and negative attitudes, suggesting a need for targeted educational campaigns to enhance booster vaccine coverage among PLWH.

However, several limitations in our study should be acknowledged: First, as with all cross-sectional studies, establishing causal relationships between independent variables and different outcomes of interest is impossible. Longitudinal studies or randomized controlled trials would be needed to examine causal relationships. Second, subjectively self-administered questionnaires may introduce recall bias, which is difficult to avoid considering the need for anonymity in our study. Future research could consider using alternative methods, such as structured interviews or electronic data collection, to minimize this bias. Third, while most of the items and scales used in this study were self-constructed based on those used in the general population, the external validation of these measures was limited. Further research should seek to validate these measures against established scales or through other external validation methods. Finally, there were variations in the distribution of sociodemographic characteristics between the two groups. Although we adjusted for these characteristics in the multivariable logistic regression model, their potential impact on the study results should be considered. Future studies could explore the potential influence of these characteristics on vaccine willingness more comprehensively and consider other statistical techniques, such as propensity score matching, to address these imbalances.

## 5. Conclusion

In conclusion, our study highlights the lower willingness of Chinese PLWH to receive a second COVID-19 booster dose compared to healthy individuals. Concerns about adverse effects and negative attitudes toward the booster dose primarily drive this reluctance. Strengthening and promoting knowledge about HIV and COVID-19 vaccination, including the importance of vaccine protection against SARS-CoV-2, is crucial. Based on the findings of this study, targeted interventions should be implemented to increase the willingness of PLWH to receive the second COVID-19 booster dose. This may include tailored education and communication strategies, providing comprehensive information and support, and engaging community resources to address the specific concerns and needs of PLWH.

## Data availability statement

The raw data supporting the conclusions of this article will be made available by the authors, without undue reservation.

## Author contributions

JX was responsible for the conceptualization of the study and funding acquisition. XL was responsible for data curation and analysis. JX, MS, SL, MY, BS, YQ, and LW were responsible for project administration and securing resources. JX and YW were responsible for supervision and reviewing and editing the article. XL and YW were responsible for writing the original draft of the paper. All authors contributed to the article and approved the submitted version.

## Funding

This work was funded by the Shenzhen Science and Technology Innovation Committee Projects (No. JCYJ20220818102817038) and the Scientific Research Foundation of Peking University Shenzhen Hospital (No. KYQD2022216).

## Conflict of interest

The authors declare that the research was conducted in the absence of any commercial or financial relationships that could be construed as a potential conflict of interest.

The reviewer XZ declared a past co-authorship with the authors YQ, MY, and JX to the handling editor.

## Publisher’s note

All claims expressed in this article are solely those of the authors and do not necessarily represent those of their affiliated organizations, or those of the publisher, the editors and the reviewers. Any product that may be evaluated in this article, or claim that may be made by its manufacturer, is not guaranteed or endorsed by the publisher.

## Abbreviations

PLWH: people living with HIV
SARS-CoV-2: severe acute respiratory syndrome coronavirus 2
COVID-19: coronavirus disease 2019
AOR: adjusted odds ratio
WHO: world health organization
HBM: health belief model
